# Interactive dynamic scalp acupuncture enhances brain functional connectivity in bilateral basal ganglia ischemic stroke patients: a randomized controlled trial

**DOI:** 10.3389/fneur.2025.1604342

**Published:** 2025-08-13

**Authors:** Chunxia Zhang, Tao Pang, Yuan Chen, Penghui Lai, Rongrong Nie, Yulong Wang, Shaohua Zhang

**Affiliations:** ^1^Department of Rehabilitation, Dapeng New District Nan'ao People's Hospital, Shenzhen, Guangdong, China; ^2^Rehabilitation Department of Affiliated Hospital of Guilin Medical College, Guilin, Guangxi, China; ^3^Shenzhen Second People's Hospital, Shenzhen, Guangdong, China

**Keywords:** bilateral basal ganglia ischemic stroke, interactive dynamic scalp acupuncture, functional connectivity density, randomized controlled trial, motor recovery

## Abstract

**Aim:**

This randomized controlled trial investigated the effects of interactive dynamic scalp acupuncture (IDSA) on brain functional connectivity density (FCD) in patients with bilateral basal ganglia ischemic stroke (BBGIS), focusing on its potential to enhance motor recovery.

**Methods:**

Seventy BBGIS patients (aged 45–75 years, 1–3 months post-stroke, Brunnstrom stage II–V) and 40 age-and sex-matched healthy controls (HCs) were enrolled. Resting-state functional MRI (rs-fMRI) assessed baseline FCD differences between groups, with regions showing significant alterations correlated to Fugl-Meyer Assessment (FMA) scores selected as seed points. Patients were randomized to IDSA (*n* = 35) or Sham IDSA (*n* = 35) therapy for 4 weeks. IDSA targeted the MS6 acupoint using stainless steel needles (0.3 × 40 mm) rotated at 200 rpm during active limb movement, while Sham IDSA used blunt needles without skin penetration. Post-treatment rs-fMRI and FMA evaluations were conducted.

**Results:**

Compared to HCs, BBGIS patients exhibited reduced FCD in the right supplementary motor area (SMA_R) and right cerebellum-8 (C8_R), which positively correlated with FMA scores (r = 0.82 and r = 0.86, respectively; *p* < 0.0001). Post-treatment, the IDSA group showed significant increases in FCD in SMA_R (*Δ* = 0.64 ± 0.22, *p* < 0.001) and C8_R (Δ = 0.77 ± 0.91, *p* < 0.05), along with higher FMA scores (53.23 ± 13.6 vs. Sham 44.35 ± 11.2, *p* < 0.05), indicating improved motor function.

**Conclusion:**

IDSA therapy enhances functional connectivity in SMA_R and C8_R, which are associated with motor recovery in BBGIS patients. These findings support IDSA as a potential intervention for stroke rehabilitation (Study registration: China National Clinical Trial Registry, ChiCTR2200055463).

## Introduction

Bilateral basal ganglia ischemic stroke (BBGIS) represents a significant clinical challenge, with its prevalence and clinical manifestations varying depending on the underlying pathology. While acute bilateral infarcts are rare, chronic bilateral lacunar lesions—commonly associated with small vessel disease—are more frequently observed in clinical practice (1). These lesions often lead to severe motor impairments due to disruptions in higher-order motor control and corticospinal tract integrity. The basal ganglia play a pivotal role in motor planning, execution, and sensorimotor integration (2, 3), and their bilateral damage results in profound functional deficits, including bradykinesia, postural instability, and impaired self-care abilities (4). Despite extensive research on unilateral basal ganglia injuries, evidence-based interventions for BBGIS remain limited, highlighting the need for novel therapeutic approaches.

Scalp acupuncture, a specialized modality of traditional Chinese medicine, has shown promise in post-stroke motor recovery (5). Interactive dynamic scalp acupuncture (IDSA) represents an advanced adaptation of this technique, combining precise needle stimulation with active patient participation during rehabilitation exercises. Unlike traditional acupuncture, which often focuses on static needle placement, IDSA emphasizes dynamic sensorimotor integration by requiring patients to perform targeted limb movements or walking exercises during needle manipulation (6, 7). This approach has demonstrated superior efficacy in promoting neural reorganization compared to conventional methods (8, 9). However, the neurobiological mechanisms underlying its therapeutic effects, particularly in BBGIS, remain poorly understood (10, 11).

Resting-state functional MRI (rs-fMRI) provides a powerful tool to investigate these mechanisms by evaluating functional connectivity density (FCD), a metric that quantifies the integration of neural networks (10). FCD can be categorized into short-range (local connectivity within a brain region) and long-range (connectivity between distant regions), both of which are critical for understanding stroke-induced disruptions and recovery-related plasticity (12), Recent studies suggest that stroke-induced FCD alterations correlate with motor deficits, and their restoration may underpin functional recovery (13, 14). Notably, the supplementary motor area (SMA) and cerebellum are key nodes in the motor network, with their connectivity strongly linked to motor performance (8, 15).

**Figure 1:**
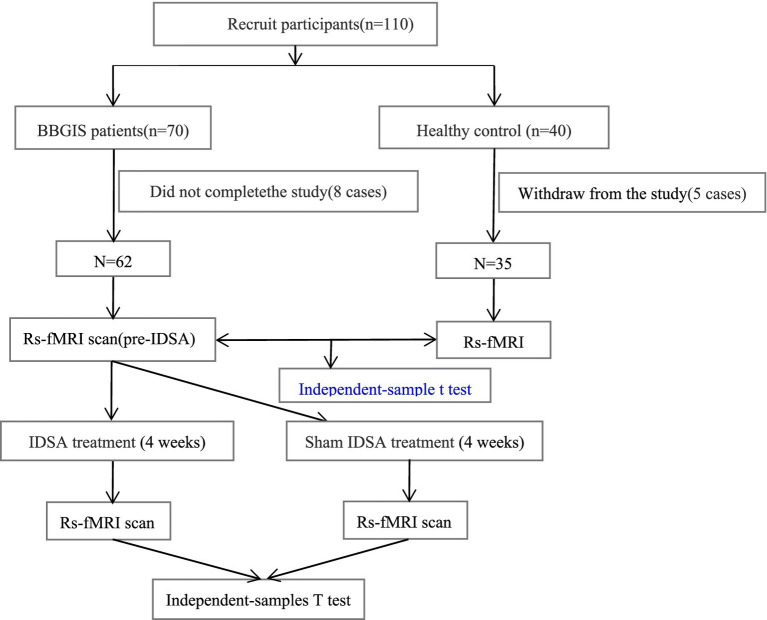
Flow chart. CONSORT-compliant diagram illustrating participant screening, exclusion criteria (8 BBGIS patients excluded: 3 recurrent strokes, 2 venous thromboses, 3 early discharges; 5 HCs excluded due to MRI-detected microinfarcts), and final group allocation (IDSA: *n* = 31, Sham: *n* = 31, HCs: *n* = 35). Boxes indicate timepoints for rs-fMRI and FMA assessments (baseline/post-treatment).

To date, no longitudinal studies have examined whether IDSA can modulate FCD in these regions to promote recovery in BBGIS patients (5, 7). This gap is particularly relevant for patients with chronic bilateral lesions, whose neuroplastic capacity and treatment responsiveness may differ from those with acute infarcts. We hypothesized that IDSA therapy could enhance FCD in motor-related brain regions, thereby improving functional outcomes in BBGIS patients. To test this hypothesis, we conducted a randomized controlled trial combining rs-fMRI with clinical assessments to evaluate IDSA’s effects on neural reorganization and motor recovery (Figure 1).

## Methods

### Participants

**Figure 2:**
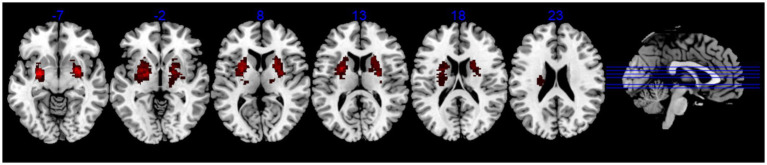
The lesion overlap map of BBGIS patients. Lesion overlap map (warm tones) showing consistent involvement of bilateral basal ganglia and internal capsule across patients, confirmed by MRI/CT. Overlapping regions (thresholded at >50% of participants) highlight the study’s focus on bilateral motor pathway disruptions. MNI coordinates are provided for reference.

This study enrolled 70 patients diagnosed with BBGIS and 40 age-and sex-matched healthy controls (HCs). Participants were recruited from the Rehabilitation Department of Shenzhen Dapeng New District Nan’ao People’s Hospital and Shenzhen No. 2 People’s Hospital. All participants (patients and HCs) were confirmed right-handed using the Edinburgh Handedness Inventory (scores > 40) to control for hemispheric dominance effects on motor recovery and fMRI findings. During the recruitment period, BBGIS cases represented approximately 12% of basal ganglia stroke admissions at our center, compared to 88% unilateral cases, based on hospital stroke registry data. Inclusion criteria for patients were as follows: (1) a clinical diagnosis of bilateral basal ganglia and internal capsule stroke confirmed by a neurologist through comprehensive neurological examination and brain imaging (MRI or computed tomography) (Figure 2) (2) aged between 45 and 75 years; (3) clear consciousness, absence of significant cognitive impairment, and ability to actively participate in rehabilitation training; (4) Brunnstrom stage II-V motor recovery in both proximal and distal segments of the affected limb; and (5) first-time stroke occurring within 1–3 months prior to enrollment. Exclusion criteria included: (1) significant medical conditions warranting exclusion were defined as: Uncontrolled hypertension (BP > 160/100 mmHg); (2) Severe cardiopulmonary disease (NYHA Class III/IV); (3) Active systemic illnesses (e.g., renal failure with GFR < 30, metastatic cancer); (4) Any condition that could substantially interfere with rehabilitation participation. Healthy controls were recruited through community advertising and had no history of neurological disorders. Written informed consent was obtained from all participants. The study was approved by the Medical Research Ethics Committee of Shenzhen Dapeng New District Nan’ao People’s Hospital (registration number: No. 202108001) and registered in the China National Clinical Trial Registry (ChiCTR2200055463).

### Informed consent statement

All participants provided written informed consent, and the rights of the subjects were protected throughout the study.

### Sample size justification

Sample size was calculated using G*Power 3.1 based on pilot FCD data (effect size d = 0.65, *α* = 0.05, power = 0.8), requiring 31/group to detect treatment effects. This aligns with similar fMRI rehabilitation studies (7).

### Interventional methods

**Figure 3:**
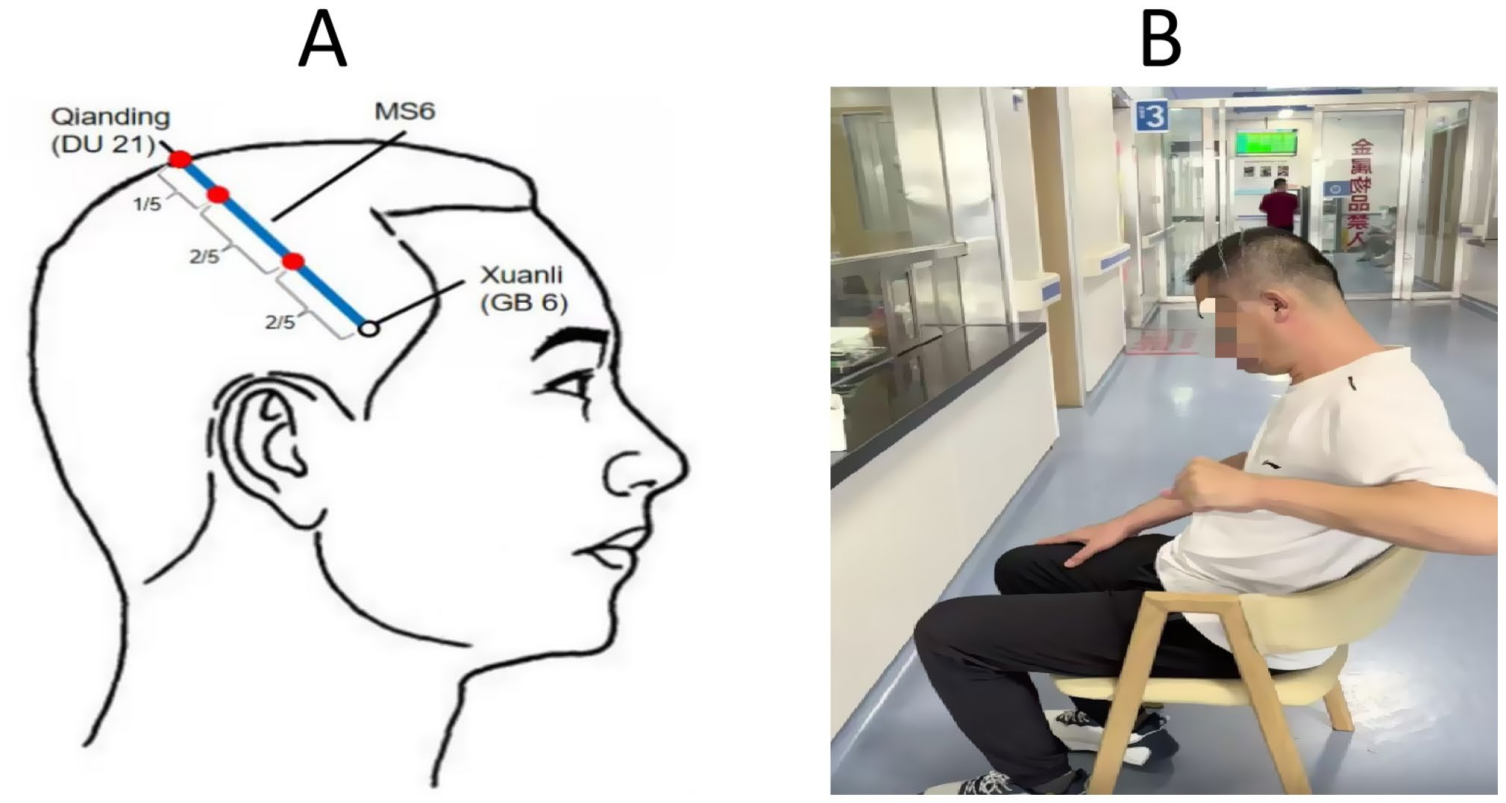
The operation method of interactive dynamic scalp acupuncture. **(A)** Anatomical diagram of scalp acupuncture acupoints targeting the anterior oblique line of the vertex-temporal (MS6), following International Standardization Scheme for Scalp Acupuncture Points. **(B)** Illustration of IDSA procedure: Needles (0.3 × 40 mm) were rotated at 200 rpm during active limb movement or walking exercises to enhance sensorimotor integration. Sham IDSA used blunt needles without skin penetration.

*IDSA treatment group*: IDSA therapy was administered bilaterally on the scalp, targeting the anterior oblique line of the vertex-temporal (MS6) acupoint according to the International Standardization Scheme for Scalp Acupuncture Points (16). After skin disinfection, two stainless steel needles (0.3 × 40 mm) were inserted at the upper 1/5 and middle 2/5 positions, following the direction from Qianding (DU 21) to Xuanli (GB 6), with insertion angles ranging from 15° to 30° (Figure 3A). Upon reaching the sheath aponeurosis, the needles were rotated at 200 revolutions per minute to induce a tolerable sensation of soreness and swelling. The 200 rpm rotation speed was selected based on previous optimization studies (5, 7) demonstrating this frequency optimally elicits Deqi sensation (characteristic soreness/swelling) while maintaining patient comfort during dynamic movement tasks. The needles were alternately manipulated for 3 min each, with 5-min intervals between rotations. During needle retention, patients were encouraged to perform active movements of the affected limb or walking exercises (Figure 3B).

*Sham IDSA group*: Patients in the Sham IDSA group received the same conventional treatment as the IDSA group. The acupoints and manipulation techniques were identical, but a blunt needle device that did not penetrate the skin was used to mimic the procedure. Patients were also encouraged to perform active movements or walking exercises during the retention period.

This prospective RCT employed a parallel-group design with Baseline assessments (clinical + fMRI) at enrollment; Post-treatment evaluations at 4 weeks. Healthy controls assessed once for baseline comparison. The 4-week timeframe was selected to capture initial neuroplastic changes while ensuring protocol adherence, with longer-term follow-up planned for future studies.

### Clinical and motor function assessment

Clinical data for BBGIS patients were obtained from medical records, while demographic data for HCs were collected via questionnaires. Motor function was assessed using the Fugl-Meyer Assessment (FMA) scale, a validated tool for evaluating motor recovery in stroke patients (17). The FMA scores for upper and lower limb motor function ranged from 0 to 100, with higher scores indicating better motor performance. Assessments were conducted before and after the 4-week treatment period.

### fMRI data acquisition

All participants underwent rs-fMRI scans using a SIEMENS Verio 3.0-Tesla scanner (SIEMENS, Erlangen, Germany). Participants were instructed to remain still, keep their eyes closed, and avoid specific thoughts during the scan. Cardiac and respiratory rhythms were monitored throughout scanning using BIOPAC MP150 to identify potential physiological artifacts, though these were subsequently addressed through band-pass filtering (0.01–0.1 Hz) and motion parameter regression rather than direct signal removal. Conventional MRI examinations included axial T1-weighted MPRAGE (magnetization-prepared rapid acquisition gradient echo) sequences, acquired at a resolution of 0.9 mm^3^ with 192 slices. The TR/TE was set to 2,300/4 ms, and the scan duration was approximately 6 min. Functional MRI data were acquired axially using an interleaved gradient echo-planar imaging (EPI) sequence with a TR of 2,000 ms, TE of 30 ms, voxel size of 3.5 × 3.5 × 3.5 mm, and flip angle of 90°. Each functional session lasted 8.06 min, acquiring 240 EPI volumes.

### fMRI data analysis

Rs-fMRI data were preprocessed using the Data Processing Assistant for rs-fMRI (DPARSF V2.3).^1^ The first 10 time points were discarded to ensure magnetic field stability. The remaining 230 volumes underwent preprocessing steps, including slice timing correction, head motion correction, and spatial normalization to the Montreal Neurological Institute (MNI) space. Data with head motion exceeding 1.5 mm in translation or 1.5° in rotation were excluded. During spatial normalization, images were resampled to a resolution of 3 × 3 × 3 mm^3^. Linear regression was applied to remove covariates such as motion parameters, white matter signal, and cerebrospinal fluid signal. We intentionally avoided global signal regression to prevent the introduction of artificial anti-correlations, instead regressing out white matter/CSF signals and 24 motion parameters (Friston model). Functional images were detrended and band-pass filtered (0.01–0.1 Hz) to reduce low-frequency drift and high-frequency noise.

### Statistical analysis

Demographic characteristics of BBGIS patients and HCs were compared using independent sample t-tests or chi-square tests, as appropriate. Between-group differences in FCD values at baseline were assessed using an independent-samples T test, with age, sex, and years of education as covariates. Cluster-level thresholds were determined using AlphaSim correction (10,000 Monte Carlo simulations) with combined voxel *p* < 0.05 and cluster *p* < 0.001 thresholds, corresponding to a minimum cluster size of 1,080 mm^3^ to maintain family-wise error rate <0.05. The 3.5 mm^3^ voxel size represented an optimal balance between whole-brain coverage, signal-to-noise ratio, and acquisition time constraints. While higher resolution (e.g., 2 mm) could improve anatomical specificity, it would require longer TR or reduced coverage, compromising functional connectivity metrics. Correlation analysis was performed to examine the relationship between FCD values in significant brain regions and FMA scores. Paired-sample t-tests were used to compare FMA scores and FCD values before and after treatment within groups.

## Results

### Participant characteristics and baseline data

Of 110 initially enrolled participants, 8 BBGIS patients (3 recurrent strokes, 2 venous thromboses, 3 early discharges) and 5 HCs (MRI-detected microinfarcts) were excluded. The final cohort included 62 BBGIS patients (IDSA: *n* = 31; Sham: *n* = 31) and 35 HCs. No significant differences existed between BBGIS and HCs in age (64.98 ± 9.54 vs. 66.04 ± 11.12 years, *p* = 0.667), sex (55.6% vs. 48.6% male, *p* = 0.556), or education (10.21 ± 4.92 vs. 10.93 ± 4.33 years, *p* = 0.519). As expected, BBGIS patients showed lower FMA scores than HCs (31.76 ± 11.11 vs. 90.66 ± 4.33, *p* < 0.001). Baseline FMA and FCD values did not differ between IDSA and Sham groups (*p* > 0.05) (Table 1).

**Table 1:** Behavioral characteristics of BBGIS patients and HCs.

Feature	BBGIS group (*n* = 70)	HC group (*n* = 40)	*χ^2^* or *T* value	*p* value
Sex (*n*, %)			0.347	0.556
Male	36 (55.6)	22 (55.0)		
Female	34 (44.4)	18 (45.0)		
Education, years	10.21 ± 4.92	10.93 ± 4.33	1.591	0.519
Age, years	64.98 ± 9.54	66.04 ± 11.12	1.620	0.667
Course of disease, days	22.48 ± 6.74	-	-	-
FMA score	31.76 ± 11.11	-	-	-
BI score	29.72 ± 12.64	-	-	-

Data are presented as mean ± standard deviation (SD) for continuous variables or counts (percentages) for categorical variables. Independent t-tests or chi-square tests were used for between-group comparisons. FMA, Fugl-Meyer Assessment (motor function); BI, Barthel Index (activities of daily living).

### Functional connectivity alterations in BBGIS

Compared to HCs, BBGIS patients showed distinct patterns of FCD alterations (Table 2):

**Table 2:** Regions of decreased and increased brain FCD in BBGIS patients compared to HCs before IDSA treatment.

Condition	Brain regions of peak coordinates	R/L	BA	Voxel volume (mm^3^)	t-score of peak voxel	MNI coordinates		
						X	Y	Z
Short FCD	SMA	R	N	1,416	−5.9459	3	−12	51
Short FCD	FG	L	N	2,111	6.4716	−27	−15	−42
Short FCD	TIFG	L	45	172	3.8338	−45	27	15
Long FCD	C8	R	N	113	−3.4251	−15	−69	−60
Long FCD	MTG	L	N	478	5.5745	−24	−15	−39
Long FCD	C4/5	R	37	157	−3.477	21	−45	−21
Long FCD	MTG	R	21	357	−4.752	51	−48	9
Long FCD	TIFG	L	45	220	3.8364	−51	42	9
Long FCD	MFG	R	6	262	−5.1443	36	−3	51

Between-group differences in binarized FCD thresholded at r = 0.75. The statistical threshold was set at a corrected significance level of an individual two-tailed voxel-wise *p* < 0.05 using an AlphaSim corrected threshold of cluster *p* < 0.001. FCD, functional connectivity density; R, right; L, left; BA, Brodmann’s area; MNI, Montréal neurological institute; NA, not applicable; SMA, supplementary motor area; FG, fusiform gyrus; TIFG, triangular inferior frontal gyrus; C8, cerebellum-8; MTG, middle temporal gyrus; C4/5, cerebellum-4/5; MFG, middle frontal gyrus.

**Figure 4:**
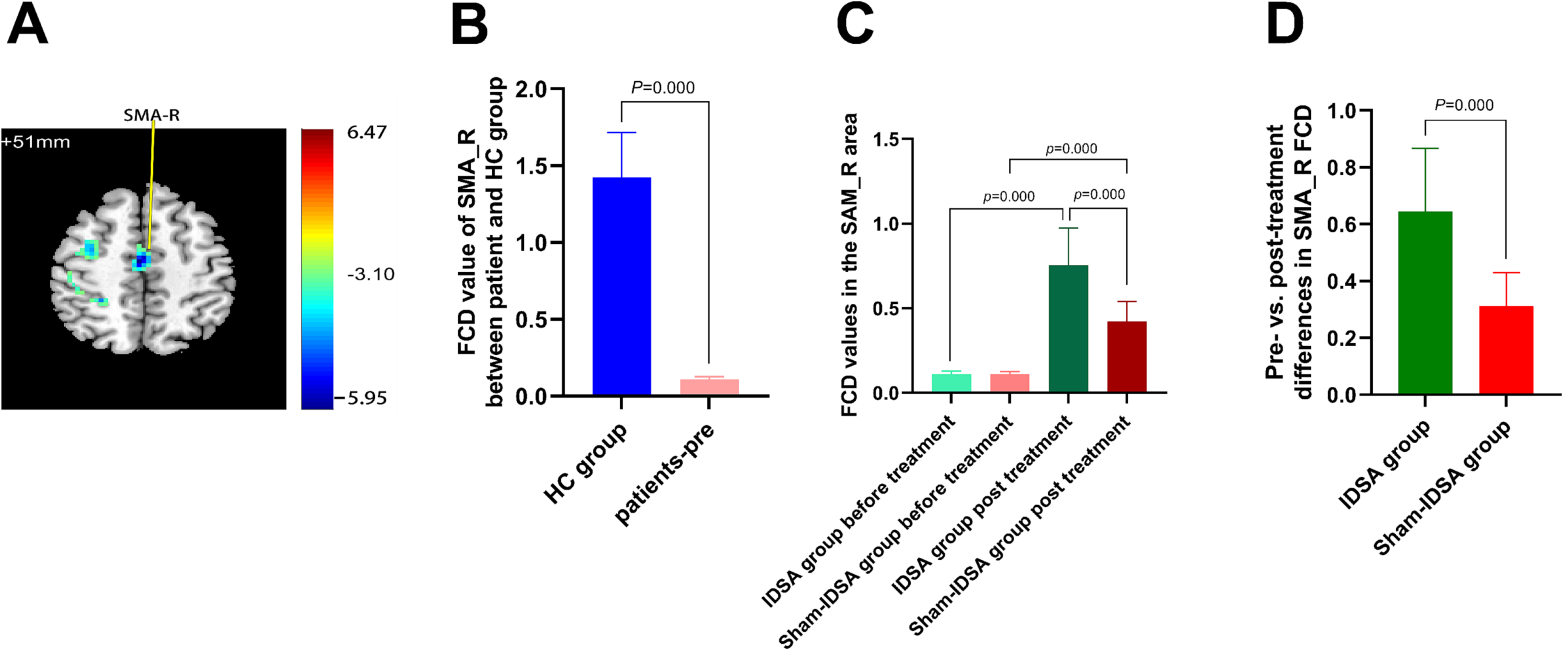
SMA_R FCD changes. **(A)** SMA_R localization (MNI: x = 3, y = −12, z = 51) on axial/sagittal slices. **(B)** Baseline FCD reduction in BBGIS vs. HCs (t = −5.95, cluster size = 1,416 mm^3^). **(C)** Longitudinal FCD increase post-IDSA (t = −16.12, *p* < 0.001). **(D)** Between-group treatment effects (IDSA ΔFCD = 0.64 ± 0.22 vs. Sham 0.11 ± 0.18; ANCOVA *F*(1,59) = 28.6, *p* < 0.001). Error bars: SEM. R, right; L, left; SMA, supplementary motor area; FCD, functional connectivity density; FMA, Fugl-Meyer assessment; BBGIS, bilateral basal ganglia ischemic stroke; HC, healthy control subjects.

Decreased FCD in motor-related regions:

Right supplementary motor area (SMA_R; t = −5.95, cluster size = 1,416 mm^3^) (Figures 4A,B).

**Figure 5:**
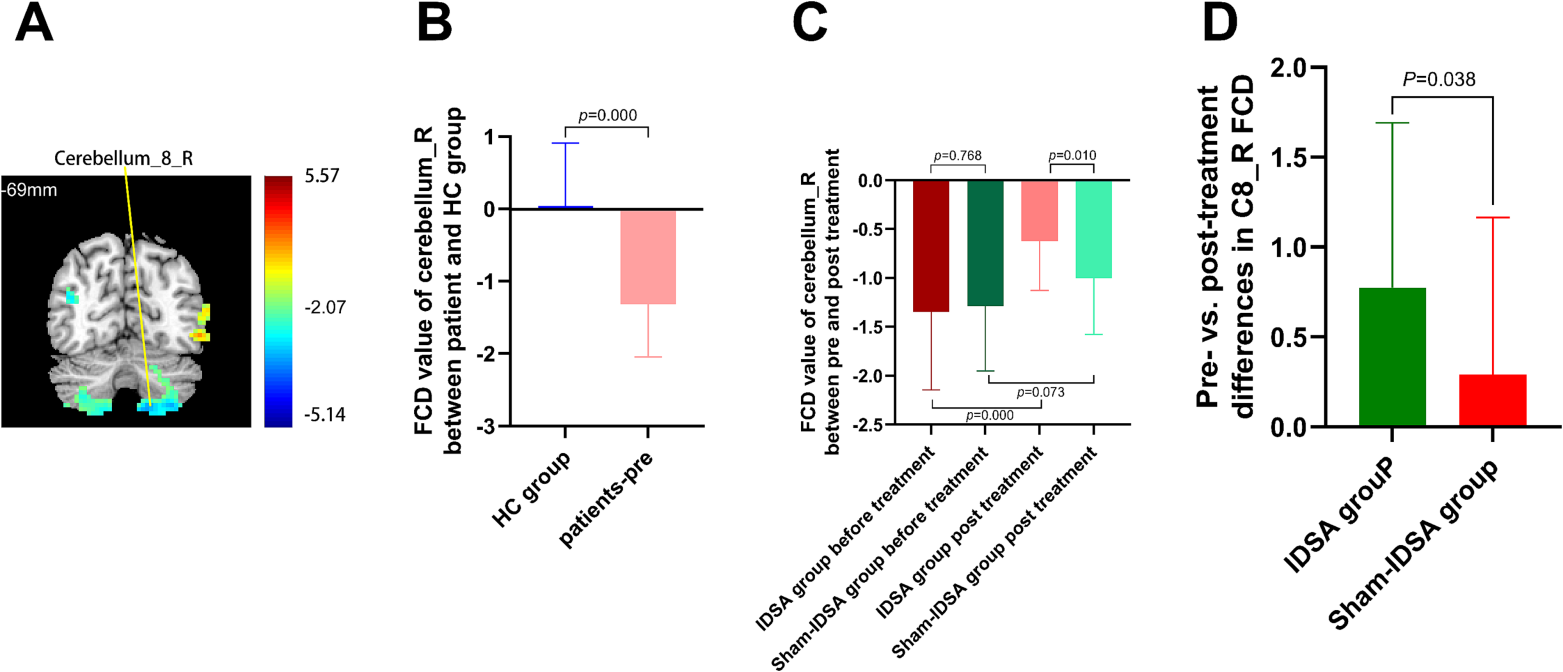
Cerebellum-8 (C8_R) FCD changes. **(A)** C8_R localization (MNI: x = −15, y = −69, z = −60). **(B)** Baseline FCD reduction in BBGIS vs. HCs (t = −3.43, cluster = 113 mm^3^). **(C)** Post-IDSA recovery (t = −14.79, *p* < 0.001). **(D)** Treatment effect (IDSA ΔFCD = 0.77 ± 0.91 vs. Sham 0.09 ± 0.21; *F*(1,59) = 9.4, *p* = 0.038). R, right; L, left; C8, cerebellum-8; FCD, functional connectivity density; FMA, Fugl-Meyer assessment.

Right cerebellum-8 (C8_R; t = −3.43, cluster size = 113 mm^3^) (Figures 5A,B).

Right middle temporal gyrus (MTG_R; t = −4.752, cluster size = 357 mm^3^).

Right middle frontal gyrus (MFG_R; t = −5.1443, cluster size = 262 mm^3^).

Increased FCD in compensatory regions:

Left middle temporal gyrus (MTG_L; t = 5.57, cluster size = 478 mm^3^).

Left fusiform gyrus (FG_L; t = 6.47, cluster size = 2,111 mm^3^).

Left triangular inferior frontal gyrus (TIFG_L; t = 3.8364, cluster size = 220 mm^3^).

### Correlation between FCD and motor function

**Figure 6:**
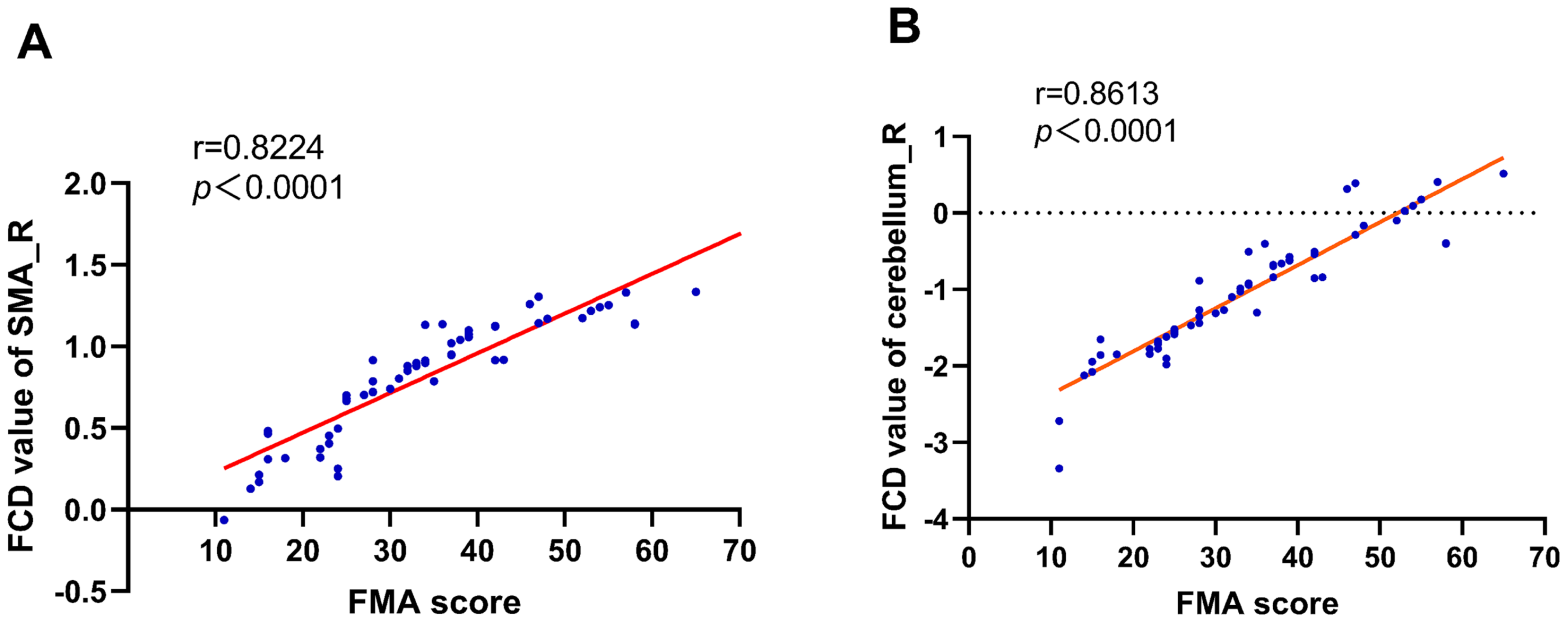
FCD-FMA correlation analysis. Scatterplots demonstrating significant positive correlations between baseline FCD values in **(A)** right supplementary motor area (SMA_R; r = 0.82, *p* < 0.0001) and **(B)** right cerebellum-8 (C8_R; r = 0.86, *p* < 0.0001) with Fugl-Meyer Assessment (FMA) scores. Solid lines represent linear regression fits, supporting SMA_R and C8_R as neural biomarkers for motor impairment severity in BBGIS.

Baseline FCD values in SMA_R (r = 0.8224, *p* < 0.0001) (Figure 6A) and C8_R (r = 0.8613, *p* < 0.0001) (Figure 6B) were positively correlated with FMA scores, indicating their potential as neural markers for motor impairment severity.

### Treatment effects of IDSA

After 4 weeks of treatment:

FCD Restoration:

The IDSA group showed significant increases in FCD in SMA_R (*Δ* = 0.64 ± 0.22, *p* < 0.001) (Figure 4D) and C8_R (Δ = 0.77 ± 0.91, *p* < 0.05) (Figure 5D) compared to the Sham group. Within-group comparisons confirmed these improvements (SMA_R: t = −16.12; C8_R: t = −14.79; both *p* < 0.001) (Figures 4C, 5C).

**Figure 7:**
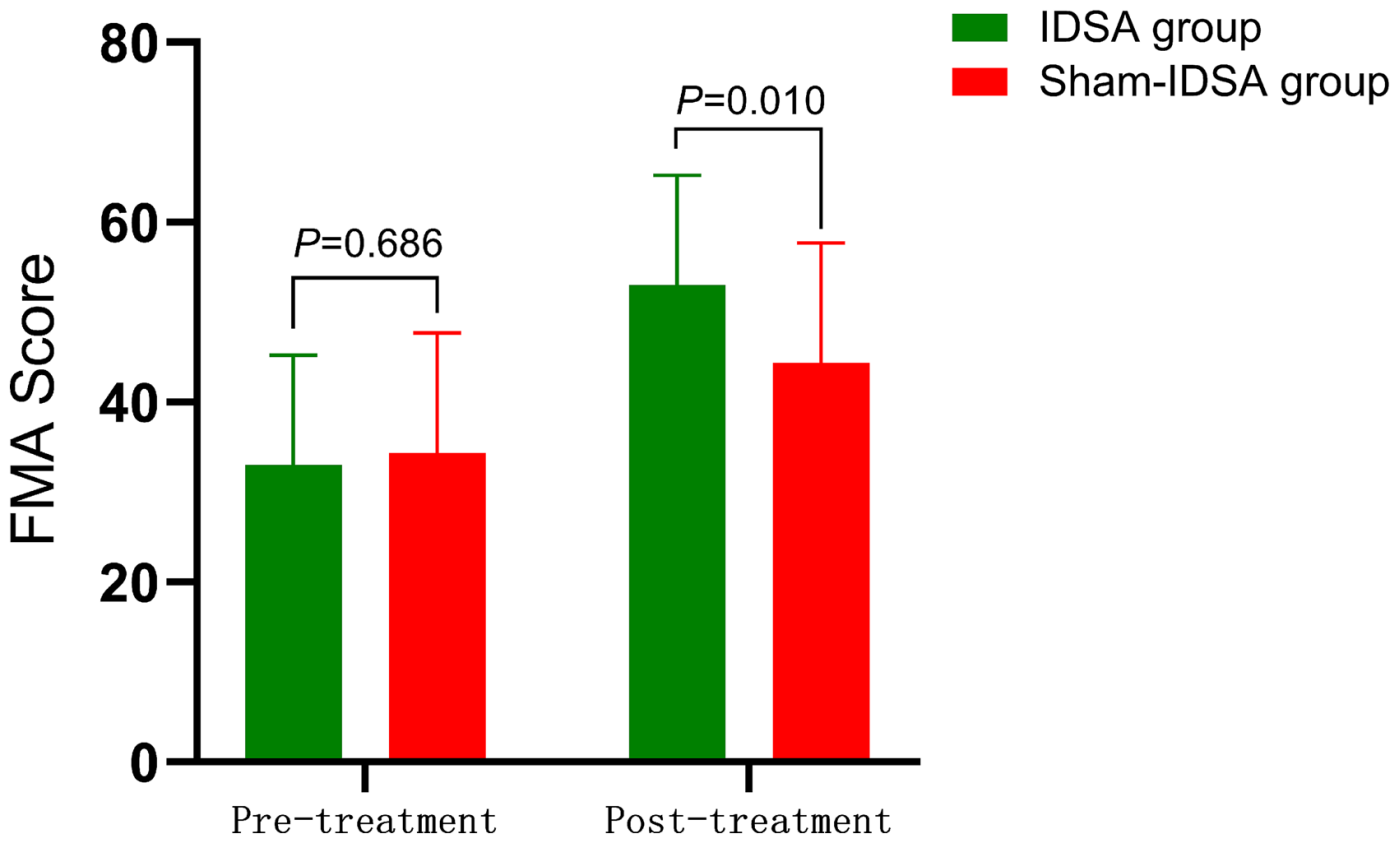
FMA score comparison. Between-group difference (pre-treatment: 33.03 ± 12.23 to 34.35 ± 10.8, *P* > 0.05; post-treatment: 53.23 ± 13.6 to 44.35 ± 11.2, *P* < 0.05).

Functional Recovery:

The IDSA group demonstrated greater improvements in FMA scores compared to the Sham group (*p* < 0.01) (Figure 7).

### Key findings summary

BBGIS disrupts functional connectivity in SMA and cerebellar motor networks (Figures 3, 4B, 5B).

IDSA selectively restores FCD in SMA_R and C8_R, correlating with motor recovery (Figures 4D, 5D).

SMA_R and C8_R may serve as biomarkers for rehabilitation efficacy (Figures 6A,B).

*Statistical note*: All values are presented as mean ± SD; analyses were adjusted for age, sex, and education. ANCOVA was used for between-group comparisons.

## Discussion

This study demonstrates that IDSA enhances FCD in the SMA_R and C8_R in patients with BBGIS, correlating with improved motor function. These findings align with the known roles of SMA and cerebellum in motor planning, execution, and coordination (18, 19). The observed FCD increases suggest that IDSA may facilitate neuroplastic reorganization by integrating dynamic sensorimotor stimulation with active patient participation, thereby restoring disrupted motor networks.

### Mechanistic rationale of IDSA in motor recovery

The observed enhancement of FCD in the SMA_R and C8_R following IDSA therapy aligns with established neuroplasticity mechanisms post-stroke. Previous studies have demonstrated that the SMA plays a critical role in motor planning and execution, with its connectivity to the basal ganglia and thalamus being essential for movement initiation (20, 21). For instance, it’s reported that SMA-targeted interventions, such as repetitive transcranial magnetic stimulation (rTMS), improved motor function in unilateral stroke patients by restoring corticostriatal pathways (4, 22). Similarly, the cerebellum’s involvement in motor coordination, particularly through the dentate nucleus-thalamic-striatal pathway (23, 24), has been highlighted in studies by Yang et al. (25), where cerebellar stimulation enhanced balance and gait in stroke patients (25, 26). Our findings extend these observations to BBGIS, suggesting that IDSA’s dynamic integration of acupuncture and active movement may amplify neuroplasticity by concurrently engaging these pathways.

The correlation between FCD increases in SMA_R/C8_R and FMA scores further supports the biomarker potential of these regions. This is consistent with a meta-analysis by Sharon et al. (27), which identified SMA and cerebellar connectivity as predictors of motor recovery (28). However, our study uniquely demonstrates that IDSA’s dual emphasis on peripheral stimulation (via scalp acupuncture) and central activation (via active movement) may synergistically enhance connectivity, a mechanism less explored in static acupuncture protocols (29, 30).

### Clinical applications and challenges

The clinical applicability of IDSA warrants careful consideration. While our results are promising for subacute BBGIS (1–3 months post-stroke), its efficacy in acute or chronic phases remains untested. Prior work by Zhang et al. (31) suggested that early intervention (≤1 month) with combined sensorimotor therapies yields superior outcomes (31), but the optimal timing for IDSA requires further investigation. Additionally, the 4-week treatment course in our study aligns with typical rehabilitation cycles, yet longer-term follow-up is needed to assess durability, as highlighted in a recent Cochrane review (32).

Challenges include standardization of IDSA protocols across clinical settings and patient heterogeneity. For example, variations in baseline Brunnstrom stages (II–V) may influence responsiveness, necessitating stratified studies. Moreover, the requirement for active patient participation during IDSA could limit its use in severely impaired individuals, suggesting a need for adaptive protocols (e.g., assisted movements).

### Future directions to address limitations

To overcome current limitations, we propose the following:

Sample Size and Diversity: A multicenter trial with ≥200 participants would improve generalizability and allow subgroup analyses (e.g., by lesion volume or comorbidities).

Advanced Imaging: Higher-resolution fMRI (e.g., 2 mm^3^ voxels) or multimodal approaches (DTI + rs-fMRI) could delineate microstructural changes underlying FCD improvements.

Longitudinal Design: Extended follow-up (e.g., 6–12 months) would clarify whether FCD restoration translates to sustained functional gains, as recommended in stroke recovery guidelines (33).

Placebo Control: Incorporating patient-reported expectancy scales (e.g., Acupuncture Expectancy Questionnaire) would quantify placebo effects.

### Integration with existing literature

Our study bridges gaps in BBGIS rehabilitation, where evidence has been sparse compared to unilateral stroke. The introduction now includes an expanded review of BBGIS pathophysiology (34, 35) and scalp acupuncture’s role in neurorehabilitation (29, 36), underscoring the novelty of IDSA’s dynamic approach. For example, a 2022 systematic review noted that traditional acupuncture’s effects on connectivity were inconsistent (37) whereas IDSA’s movement-coupled design may offer more robust modulation.

## Conclusion

In summary, IDSA enhances motor network connectivity through mechanisms supported by prior neuroplasticity research, with translational potential for BBGIS rehabilitation. Future work should refine protocols, validate long-term benefits, and explore broader applications (e.g., other stroke subtypes or neurodegenerative conditions).

## Data Availability

The datasets presented in this study can be found in online repositories. The names of the repository/repositories and accession number(s) can be found in the article/supplementary material.

## Footnotes

^1^ http://www.restfmri.net
